# Ultrasound Image Classification of Thyroid Nodules Based on Deep Learning

**DOI:** 10.3389/fonc.2022.905955

**Published:** 2022-07-15

**Authors:** Jingya Yang, Xiaoli Shi, Bing Wang, Wenjing Qiu, Geng Tian, Xudong Wang, Peizhen Wang, Jiasheng Yang

**Affiliations:** ^1^ School of Electrical & Information Engineering, Anhui University of Technology, Ma’anshan, China; ^2^ Scientific System, Geneis Beijing Co., Ltd., Beijing, China; ^3^ Qingdao Genesis Institute of Big Data Mining and Precision Medicine, Qingdao, China

**Keywords:** thyroid nodule, ultrasound images, deep learning, convolutional neural network, Grad-CAM, feature extraction

## Abstract

A thyroid nodule, which is defined as abnormal growth of thyroid cells, indicates excessive iodine intake, thyroid degeneration, inflammation, and other diseases. Although thyroid nodules are always non-malignant, the malignancy likelihood of a thyroid nodule grows steadily every year. In order to reduce the burden on doctors and avoid unnecessary fine needle aspiration (FNA) and surgical resection, various studies have been done to diagnose thyroid nodules through deep-learning-based image recognition analysis. In this study, to predict the benign and malignant thyroid nodules accurately, a novel deep learning framework is proposed. Five hundred eight ultrasound images were collected from the Third Hospital of Hebei Medical University in China for model training and validation. First, a ResNet18 model, pretrained on ImageNet, was trained by an ultrasound image dataset, and a random sampling of training dataset was applied 10 times to avoid accidental errors. The results show that our model has a good performance, the average area under curve (AUC) of 10 times is 0.997, the average accuracy is 0.984, the average recall is 0.978, the average precision is 0.939, and the average F1 score is 0.957. Second, Gradient-weighted Class Activation Mapping (Grad-CAM) was proposed to highlight sensitive regions in an ultrasound image during the learning process. Grad-CAM is able to extract the sensitive regions and analyze their shape features. Based on the results, there are obvious differences between benign and malignant thyroid nodules; therefore, shape features of the sensitive regions are helpful in diagnosis to a great extent. Overall, the proposed model demonstrated the feasibility of employing deep learning and ultrasound images to estimate benign and malignant thyroid nodules.

## Introduction

Thyroid nodules can be divided into benign and malignant nodules. According to research, the incidence of thyroid cancer has increased by 2.4 times in the past 30 years, which is one of the fastest-growing malignant tumors (1–4).

There are two ways to diagnose thyroid nodules, fine needle aspiration (FNA) and ultrasound. FNA is the gold standard for thyroid nodule detection, but it is traumatic to the human body (5). Acharya et al. indicated that 70% of the diagnoses with FNA were benign; about 4% (1% to 10%) were malignant or suspected malignant (6). Therefore, FNA is not suitable for all thyroid nodules. Ultrasound is a non-invasive and radiation-free method, which has low cost and short time and can even show a few millimeters of lesions, so it has been widely used (7, 8). However, the diagnosis of ultrasound depends on the experience and judgment of doctors to a great extent, which may lead to misdiagnosis (9). The computer-aided diagnosis (CAD) system could help doctors diagnose thyroid nodules objectively (10–14). Park et al. confirmed that CAD and radiology were generally comparable; CAD is feasible to assist doctors in diagnosis (15). Chang et al. extracted six features and calculated the F-score of these feature sets and screened out the main texture features through support vector machines (SVMs) for subsequent classification (16). Lyra et al. adopted the gray-level co-occurrence matrix (GLCM) to characterize texture features of thyroid nodules (17). Keramidas et al. tried to classify thyroid nodules by local binary patterns (LBPs) (18). Acharya et al. segmented thyroid nodule images manually, extracted four texture features from images, including fractal dimension (FD), LBP, Fourier spectrum descriptor (FS), and Laws’ texture energy (LTE), and then used these feature vectors to predict thyroid nodules using SVM, decision tree (DT), Sugeno fuzzy, gaussian mixture model (GMM), K-nearest neighbor (KNN), radial basis probabilistic neural network (RBPNN), and naive bayes classifier (NBC) (19). Ma et al. tried to classify thyroid nodules by five different machine learning methods, namely, deep neural network (DNN), SVM, central clustering methods, KNN, and logistic regression; the accuracy was 0.87 (20).

These methods need to extract and even fuse many features manually to achieve high results. While deep learning can deal with massive data and learn deeper and more abstract features automatically, it also avoids complex manual feature extraction (21–23). Guan et al. employed the Inception v3 model to classify thyroid nodules (24). Chi et al. proposed a deep learning framework to extract features from ultrasound images. The proposed model achieved 96.34% accuracy, 86% sensitivity, and 99% specificity on their own database (25). Peng et al. developed a ThyNet model to classify thyroid nodules from multiple hospitals; the area under the receiver operating characteristic curve (AUROC) reached 0.922, and the AUROC of the radiologist’s diagnosis improved from 0.837 to 0.875 with the aid of ThyNet, and from 0.862 to 0.873 in the clinical test. The frequency of FNA is reduced in the simulated scenario, while the missed diagnosis of malignant tumors was reduced from 18.9% to 17% as well (26). Avola et al. proposed a knowledge-driven classification framework; the proposed framework finally achieved an AUC value of 98.79% (27). Ye et al. applied a residual network pretrained by transfer learning to classify thyroid nodules; the highest accuracy reached 93.75% (28). Ma et al. tried to classify thyroid nodules by two pretrained convolutional neural networks (CNNs); the feature maps obtained by the two CNNs were fused (29). Sun et al. transferred the CNN model learned from ImageNet as a pretrained feature extractor to a new dataset of ultrasound images. The proposed method combined traditional low-level features extracted from the histogram of oriented gradient (HOG) and LBP with high-level deep features extracted from CNN models to form a hybrid feature space. The experimental accuracy was 93.10% (30). Furthermore, Chen et al. used a deep learning ultrasound text classifier for predicting thyroid nodules. The method achieved 93% and 95% accuracy on real medical datasets and standard datasets, respectively (31).

In this paper, 508 ultrasound images were collected from the Third Hospital of Hebei Medical University in China, which are the same as that by Ma et al. (20). The main work is as follows. First, the ResNet18 model, combined with transfer learning, was employed to classify benign and malignant thyroid nodules (32). Second, a heatmap was used to visualize the model’s attention on thyroid nodule images. Next, the highlighted regions expressed by heatmaps were extracted and analyzed from original images. Finally, we found that the characteristics of thyroid nodules with different properties were quite different (P < 0.05 means the difference is statistically significant.). In summary, our method is effective in the classification of thyroid nodule images.

## Material and Method

### Ultrasound Image Data

In this paper, we used the ultrasound images of thyroid nodules provided by the Third Hospital of Hebei Medical University in China. Images of thyroid nodules had been checked by an experienced doctor; artifacts and blurred images were excluded. The pathologist determined the benign and malignant nodules according to the pathological diagnosis. Finally, we collected 508 ultrasound images, of which 415 were benign nodules and 93 were malignant nodules. Each ultrasound image corresponded to a patient. Among them, 70% of the dataset served as the training set and 30% as the test set. There was no overlap between the training and testing sets. **Table 1** shows the distribution of benign and malignant nodules in the training and testing groups.

**Table 1:** The distribution of thyroid nodules in the training and testing groups.

Dataset	Benign	Malignant	Total
Train	291	66	357
Test	124	27	151
Total	415	93	508

**Figure 1:**
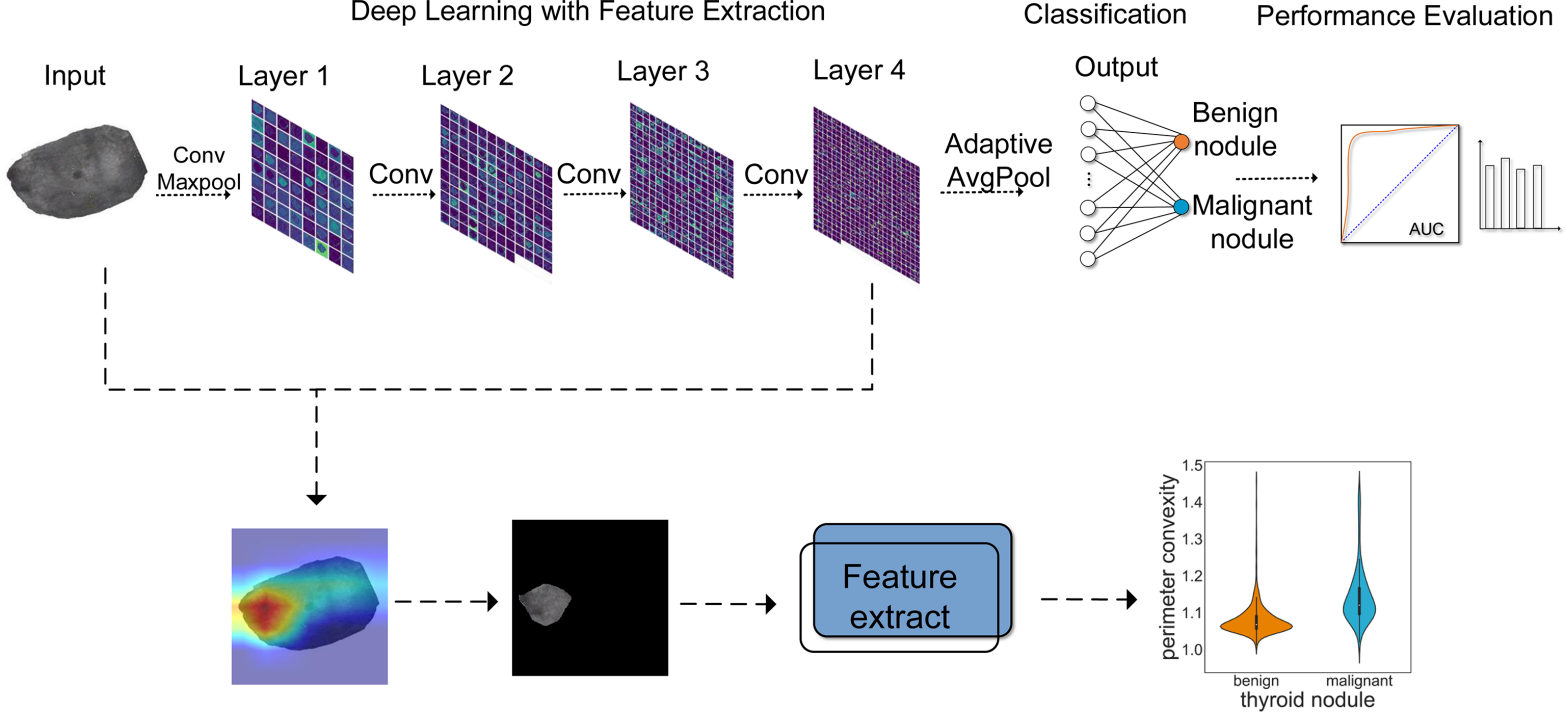
The workflow for thyroid module classification with ResNet18. Layer 1~layer 4 show the process of image analysis by ResNet18. With the increase of layers, the features extracted by the model became more abstract. AUC and other evaluation indicators were used to evaluate the effect of model classification. In addition, a heatmap was employed to visualize the prediction results, by which we extracted and analyzed the highlighted areas.

The complete process of predicting benign and malignant thyroid nodules is shown in **Figure 1**, which can be divided into three components. The Resnet18 model was employed to diagnose whether the nodule is benign or malignant; the heatmap shows the highlighted regions of the model, which were then extracted and analyzed.

### A Model for Predicting Thyroid Nodules With Ultrasound Images

**Figure 2:**
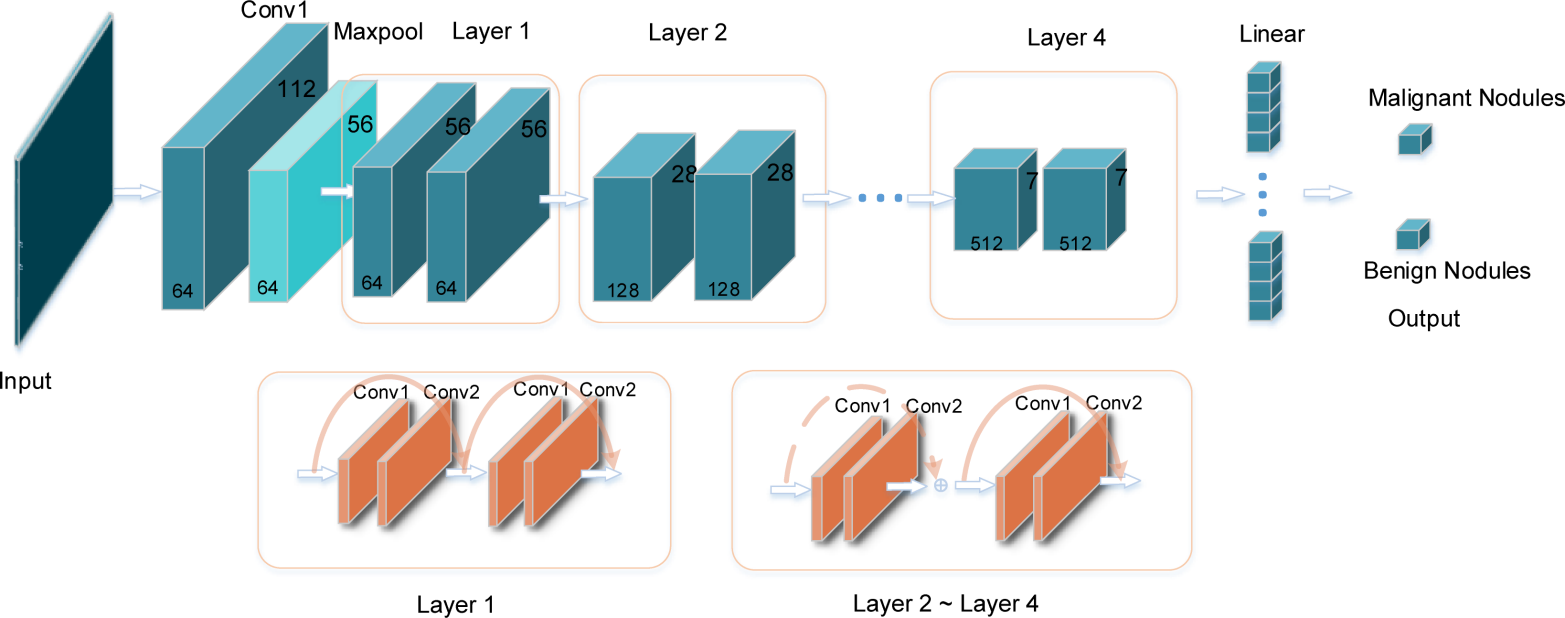
The specific structure of the ResNet18 model. The input is the image of thyroid nodules with the same size. After the convolution layers and pooling layers, the image features are extracted automatically. The output layer is the result of classification: benign or malignant nodule.

The convolution neural network performs well in image classification. The CNN has an input layer, hidden layers, and an output layer. With the image always as input, the hidden layers are used to extract features in the image. ResNet is a classical convolutional neural network, which was proposed by He et al. (32); it was the champion at the 2015 ImageNet Large Scale Visual Recognition Challenge (ILSVRC). The structure of the ResNet18 network is shown in **Figure 2**. A 7*7 convolution and a 3*3 max pooling operation were employed successively. Then it was followed by four layers, with each layer containing two basic blocks, and each basic block containing two convolutions, a batch normalization, an activation function, and a shortcut connection. Then it was modified to two classes. Among them, the role of batch normalization is to speed up the training and convergence of the network, preventing the gradient from vanishing, exploding, and overfitting at the same time. The activation function adds non-linear factors to improve the expressive ability of the neural network. The shortcut connection directly bypasses the input information to the output; this ensures the integrity of information transmission. The constructure of layers is depicted in **Figure 2**; the dotted line indicates that the number of channels has changed, and the solid line indicates that the number of channels has not changed.

Although ResNet18 predicts well in natural images, the results cannot be guaranteed to be optimal due to the huge difference between ultrasonic images and natural images. In addition, it is very complicated to train the neural network from scratch, which needs a lot of computing and memory resources. Transfer learning extracts basic information from the source dataset and applies it to the target domain by fine-tuning parameters. It can use fewer computing resources and shorter time to train the model to obtain better results.

Cross entropy (CE) is a commonly used loss function. We can obtain results that are consistent with expectations by using cross entropy as the loss function and minimizing the target loss function as the goal. Unfortunately, our dataset is unbalanced. If CE is used as the objective function, different categories will be given equal weights when calculating, which may greatly interfere with the learning process of model parameters. Many negative samples constitute a large part of the loss, thus controlling the direction of gradient updates, making the final trained model more inclined to classify samples into this type. Therefore, in order to avoid the hidden dangers that may be caused by dataset imbalance, focal loss was employed (33). Focal loss was proposed by Lin et al. in 2017; it was applied to solve the problem of data imbalance and difficult samples. Focal loss is derived from the CE loss function; CE is defined as:


(1) $$\begin{array}{l} CE\left(p,y\right)=\{\begin{array}{l} -log\left(p\right),\;\\ -log\left(1-p\right),\; \end{array}\;\begin{array}{r} if\;y=1\\ otherwise \end{array} \end{array}$$

where y represents the labels of negative and positive samples, corresponding to 0 or 1 in binary classification, and p ∈ [0,1] represents the estimated probability of the class labeled y = 1.


(2) $$p_{t}=\{\begin{array}{l} p,\;\\ 1-p, \end{array}\;\begin{array}{r} if\;y=1\\ otherwise \end{array}$$

At this point, CE becomes:


(3) $$CE\left(p,y\right)=-log\left(p_{t}\right)$$

Lin et al. added a modulating factor on the basis of CE: (1−*p*_*t*_)^*γ*^; α is the adjustment factor. Finally, focal loss can be written as:


(4) $$FL\left(p_{t}\right)=-\alpha_{t}{\left(1-p_{t}\right)}^{\gamma }log\left(p_{t}\right)$$

In summary, our framework consists of the following steps.

1. Assign a label for each thyroid nodule image.
2. Resize input images to 224*224, then transform them by flip, rotate, and so on to generate a more complex and diverse dataset, then normalize images to improve the accuracy and generality.
3. Pretrain the ResNet18 model on ImageNet to learn general image feature parameters from natural images.
4. Fine-tune the pretrained model, and adjust the 1000 classification on ImageNet to 2 in our dataset.
5. Adjust the parameters about the network, set the learning rate to 0.0001, and employ the Adam optimizer and focal loss function to optimize the network.
6. Perform pooling and fully connect the layers, then output the prediction results.

### Visualizing the Highlighted Regions by Heatmap

CNN’s performance is excellent, but it sacrifices intuition and interpretability, and it is hard to interpret the prediction results of the model. Class activation mapping (CAM) is a modern technique for model interpretation (34). However, the disadvantage is that CAM depends on the global average pooling layer; if not, we need to change and retrain the model. Therefore, the use of CAM is not convenient. Grad-CAM alleviates this problem (35). It does not need to modify the structure of the existing model, which makes it applicable for any CNN-based architecture. Grad-CAM shows the contribution distribution of the model output by heatmap, and the contribution is shown by colors; the red color in the heatmap represents a large contribution to the output, which is the main basis for judgment, while blue represents a small contribution. As shown in formula (5), let y be the probability of class and ∂_*k*_ be the feature map of the last convolutional layer of the network. Compute the gradient of y with respect to ∂_*k*_ and take a global average pooling of all pixels (i and j are width and height, respectively) to obtain a weight ∂_*k*_, which represents the importance of feature map k to discriminate the thyroid nodule category.


(5) $$\partial_{k}\;=\frac{1}{Z}\;\sum_{ij}\frac{\partial y}{\partial A_{ij}^{k}}$$

Next, weighted combinations were performed to sum the feature maps. Then, a ReLU function was followed, because we only focus on the areas that have a positive impact on the class judgment, as described in formula (6). Grad-CAM shows the areas of positive impact clearly by the heatmap.


(6) $$L_{Grad-CAM}\;=ReLU\left(\Sigma_{k}\partial_{k}A^{k}\right)$$

### Extracting and Analyzing the Highlighted Regions in Heatmaps

After visualizing the area of interest of the model by Grad-CAM, the highlighted regions based on the heatmap were extracted. First of all, according to the corresponding relationship between HSV and RGB colors, the image was converted from RGB to HSV color space. Then, the red areas in the heatmap were captured; we extracted the boundaries of the red area and overlapped them with the original image. Then the extracted areas were analyzed.

In general, image feature extraction includes shape, texture, and color feature extraction. These features depict images from different aspects. Shape features are mostly used to describe shapes of objects in images (36–38). Combined with the extracted areas of thyroid nodules, the form parameter, area convexity, and perimeter convexity were adopted to describe the shape. These features might not be the most perfect representation of the properties of the target regions but may be the most appropriate description of the shape of the regions.

The form parameter refers to the ratio of the area to the square of perimeter in one region, which indicates the complexity of the edge. The formula of the form parameter is as follows:


(7) $$F\;=\;\frac{4*pi*S}{P^{2}}$$

where S represents the area of the region and P represents the perimeter of the region. A smaller value for the form parameter indicates a more complex edge of the region.

The area convexity is the ratio of the area to the convex hull area. The convex hull refers to the smallest convex polygon containing the specified area; that is to say, all points in the target region are on or inside the surrounding convex hull area. The formula for area convexity is as follows.


(8) $$S\;=\;\frac{S}{S_{ch}}$$

*S_ch_* represents the convex hull area. The area convexity is smaller than or equal to 1; the smaller the value is, the more complex the edge of the region is.

Similarly, the perimeter convexity refers to the ratio of the perimeter to the convex hull perimeter of the region.


(9) $$P\;=\;\frac{P}{P_{ch}}$$

where *P_ch_* represents the perimeter of the convex hull, and the perimeter convexity is equal to or larger than 1. The larger the value, the more complex the edge of the region.

### Evaluation Criteria

Our model was evaluated by ROC curve, accuracy, recall, precision, and F1 score (39, 40), which are defined as follows:


(10) $$Accuracy=\frac{TP+TN}{TP+FN+TN+FP}$$

(11) $$Recall=\frac{TP}{TP+FN}$$

(12) $$Precision=\frac{TP}{TP+FP}$$

(13) $$F1\;score=\frac{2\;*\;Precision\;*\;Recall}{Precision+Recall}$$

where TP, TN, FP, and FN represent true positive, true negative, false positive, and false negative, respectively.

## Results

### Our Model Performs Better Than Existing Methods

**Figure 3:**
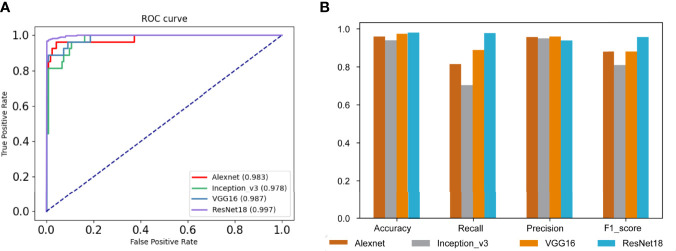
Evaluations of model results. **(A)** The receiver operating characteristic (ROC) curves and the area under the curve (AUC) of our model and other comparative models. **(B)** The performance of our model and other comparative models on accuracy, recall, precision, and F1 score.

Our dataset was randomly divided into 10 times, and we calculated the average of the 10 times to avoid the chance of the results caused by one division. Compared with AlexNet, Inception_v3, and VGG16, the performance of our model was best, the AUC was 0.997, and as shown in **Figure 3** **A**, the average accuracy, recall, precision, and F1 score were 0.984, 0.978, 0.997, and 0.957, respectively; our model performed well in the above indicators. The results are shown in **Figure 3** **B**.

### Visualizing With Grad-CAM

**Figure 4:**
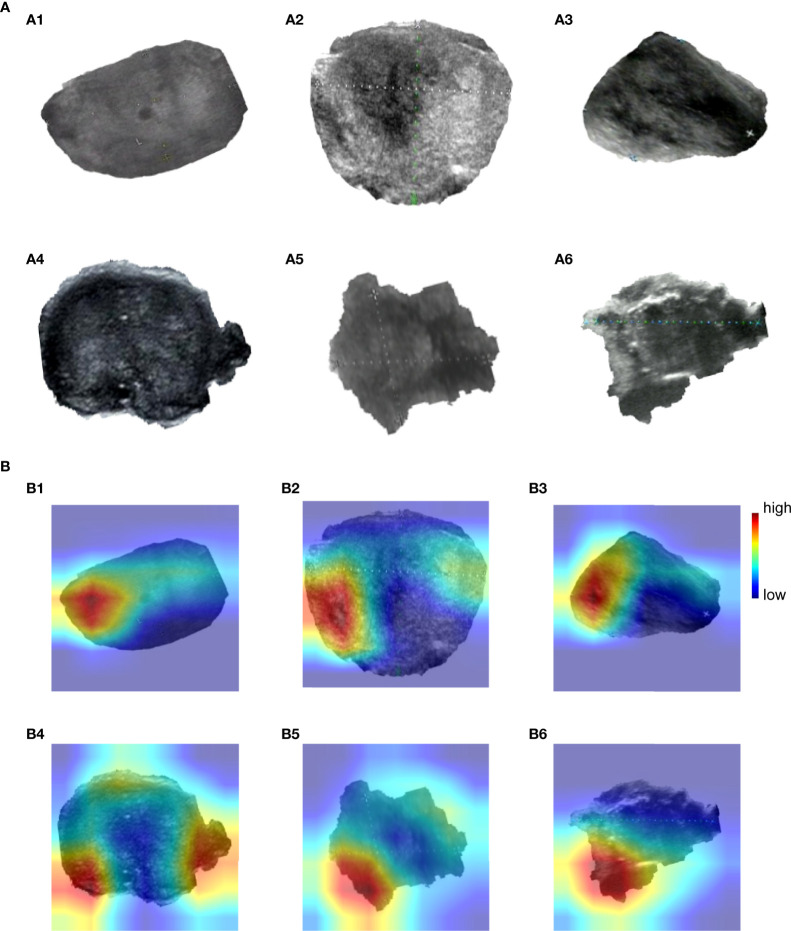
Grad-CAM visualizes highlighted regions. **(A)** (a1)~(a6) are original images, (a1)~(a3) are benign nodules, (a4)~(a6) are malignant nodules. **(B)** (b1)~(b6) are heatmaps drawn by Grad-CAM, corresponding to **Figure 4** (a1)~(a6).

As we mentioned in the “Visualizing the highlighted regions by heatmap” section, the model achieved unprecedented accuracy in image classification, but the interpretability was poor. Visualization is helpful to understand and debug the model. Grad-CAM was used to validate model predictions on images; it adopted the final convolutional layer gradients to generate the positioning heatmaps when predicting. **Figure 4** **A** shows original images, where (a1)~(a3) are benign nodules and (a4)~(a6) are malignant nodule images. **Figure 4** **B**) shows the corresponding heatmaps by Grad-CAM. The highlighted regions in the heatmaps are shown in red, and the weak regions are shown in blue. The red and blue marks represent the regions of strong and weak emphases, respectively.

### Region Extraction and Analysis

**Figure 5:**
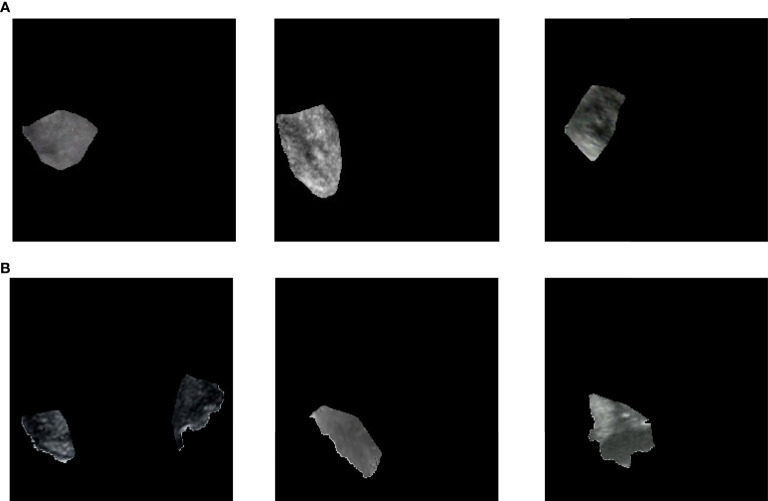
Extract highlighted regions in heatmaps. These heatmaps correspond to **Figure 4A**) or **(B)**. Samples in **(A)** are the extraction result of benign thyroid nodules, which correspond to **Figure 4** (A1–A3) or (B1–B3). Samples in **(B)** are the extraction result of malignant thyroid nodules, which correspond to **Figure 4** (a4)~(a6) or (b4)~(b6).

**Figure 6:**
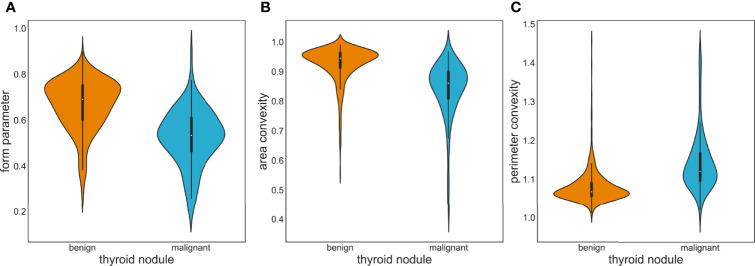
Violin plots of image feature distribution with benign and malignant nodules. **(A)** Form parameter. **(B)** Area convexity. **(C)** Perimeter convexity.

OpenCV was used to perform region extraction on the red highlight in the heatmap. The extracted results are shown in **Figure 5**; samples in (a) are the extraction result of benign thyroid nodules, and those in (b) are malignant. We found that the outline boundary of benign nodules was relatively regular, while the outline boundary of malignant nodules was relatively irregular. To verify whether almost all thyroid nodules fit this phenomenon, the shape features of thyroid nodules were calculated according to the above formulas. As a result, the form parameter, area convexity, and perimeter convexity were statistically different for benign and malignant nodules (their p-values were 1.68e-27, 7.01e-32, and 8.1e-33, respectively. P < 0.05 means the difference is statistically significant). As shown in **Figure 6**, violin plots were employed to describe the distribution of values of benign and malignant thyroid nodules with the abovementioned features.

## Discussion

The prevalence of thyroid nodules is increasing year by year, and people’s awareness of health management is also gradually improving. As a result, the burden of ultrasound doctors in hospitals and physical examination institutions is increasing. If an AI-assisted diagnosis system can be used to assist doctors to distinguish ultrasound image data, the pressure of doctors will be relieved and work efficiency will be improved. Moreover, the interpretation of ultrasound images largely depends on the clinical experience of radiologists. It was reported that the sensitivity of radiologists varied from 40.3% to 100%, and the specificity from 50% to 100% (41–44). Computer-aided diagnosis can provide more objective and more accurate results, which is very helpful to doctors with less experience. Grani et al. showed that many thyroid nodules removed by surgery were not malignant, which increased the economic burden and physical pain for patients (45), whereas the AI-assisted diagnosis system based on deep learning algorithms could lower the false positive rate and then help to reduce unnecessary FNAB and surgery.

In this paper, the ResNet18 framework was applied to train the model and Grad-CAM was proposed to highlight sensitive regions in the ultrasound images. Finally, the 10-time average AUC of our proposed method was 0.997, and the average accuracy was 0.984, which is higher than the accuracy of 0.89 designed by Ma et al. (20). Moreover, the shape features of the sensitive regions rather than other features are more helpful in the discrimination of benign and malignant tumors. From the perspective of methodology, the performance of neural network-based methods is generally higher than the traditional feature-based methods. CNNs can learn efficient and useful features automatically, avoiding the time-consuming and laborious task of obtaining features manually.

Although the proposed method achieved a supportive result, it still had some limitations. Firstly, the number of images for training and testing is insufficient and multicenter data are not available. In the future work, we will collect more data to validate the model performance. Secondly, a more detailed classification of thyroid nodules may be tried using a variety of algorithms as benign thyroid nodules can be divided into benign follicular nodules and follicular adenomas, etc., clinically, and malignant thyroid nodules can be divided into papillary, follicular, etc., medullary carcinomas. Finally, yet importantly, the results were obtained based on static ultrasound images and we should consider how to better assist doctors in making decisions in a real clinical environment.

It is believed that deep learning in diagnosing thyroid nodules has a bright future. Deep learning algorithms have been widely concerned and applied in various fields. They can map unstructured information to structured forms and learn relevant information automatically. The automatic and intelligent method not only improves the efficiency of diagnosis but also ensures the reliability, which may significantly affect early diagnosis and subsequent treatment.

## Conclusion

In this paper, we have explored the problem of thyroid nodule classification. ResNet18 was deployed with 508 thyroid nodule ultrasound images. Due to insufficient datasets, transfer learning was adopted. At the same time, considering the imbalance of the dataset, focal loss was employed to adjust the weight of data. Finally, the AUC was 0.997, which means that we can predict almost all thyroid nodules correctly. Moreover, in order to visualize the model’s attention in thyroid nodule images and help understand the model’s predictions more easily, Grad-CAM was used to identify sensitive regions in the learning process of ultrasound images, which were an important reference of image prediction. The regions concerned by the model were segmented and analyzed. Finally, in the region of interest, there are differences between benign and malignant nodules. The results of this study show that our model could diagnose well benign and malignant thyroid nodules in ultrasound images. Besides, our localization information could be regarded as a second opinion for clinical decision-making. The proposed method could assist doctors in making better decisions, reducing the time for human participation, and improving the efficiency of diagnosis.

## Data Availability Statement

The original contributions presented in the study are included in the article/supplementary material. Further inquiries can be directed to the corresponding authors.

## Author Contributions

PW and JSY designed the study; JYY, XS, BW, WQ, and GT performed the study, analyzed the data, and interpreted the data; JYY and JSY wrote the manuscript; BW, JYY, GT, and PW reviewed the manuscript. All authors contributed to the article and approved the submitted version.

## Funding

This work was supported by the National Natural Science Foundation of China (Nos. 51574004, 62172004) and Natural Science Foundation of the Higher Education Institutions of Anhui Province, China (Nos. KJ2019A0085, KJ2019ZD05).

## Conflict of Interest

JYY, XS, WQ, and GT are currently employed in Geneis Beijing Co., Ltd.

The remaining author declares that the research was conducted in the absence of any commercial or financial relationships that could be construed as a potential conflict of interest.

## Publisher’s Note

All claims expressed in this article are solely those of the authors and do not necessarily represent those of their affiliated organizations, or those of the publisher, the editors and the reviewers. Any product that may be evaluated in this article, or claim that may be made by its manufacturer, is not guaranteed or endorsed by the publisher.
